# Understanding diverse TRPV1 signaling – an update

**DOI:** 10.12688/f1000research.20795.1

**Published:** 2019-11-25

**Authors:** Michael C. Andresen

**Affiliations:** 1 Department of Physiology and Pharmacology, Oregon Health & Science University, Portland, OR, 97239, USA

**Keywords:** TRPV1, NTS, solitary tract nucleus, synaptic transmission, vagus, cranial nerve, visceral afferent

## Abstract

The transient receptor potential vanilloid 1 (TRPV1) is densely expressed in spinal sensory neurons as well as in cranial sensory neurons, including their central terminal endings. Recent work in the less familiar cranial sensory neurons, despite their many similarities with spinal sensory neurons, suggest that TRPV1 acts as a calcium channel to release a discrete population of synaptic vesicles. The modular and independent regulation of release offers new questions about nanodomain organization of release and selective actions of G protein–coupled receptors.

That burning sensation in our mouth and throat on tasting a piquant food containing capsaicin introduces us to the transient receptor potential vanilloid 1 (TRPV1). This burning sensation relies on activation of TRPV1 channels expressed in local primary sensory neurons and, when that message arrives in the cortex, it is perceived as irritation or pain. Capsaicin has had a long culinary and experimental history, but the cloning of TRPV1 defined a major breakthrough by shifting the focus to its mechanism of action ^1^. TRPV1 is a homotetrameric membrane protein combining a transmembrane pore with domains devoted to vanilloid, thermal, and proton stimuli—each capable of triggering channel gating ^2^. This single protein provides integral, polymodal transduction via ion channel activation. Discovery of TRPV1 gave structure and focus to a varied and often confusing experimentation history with natural substances such as capsaicin, which showed a mix of response activation with response inhibition ^3, 4^ plus a rich cultural history reaching to pre-Columbian times ^5^. TRPV1 has come to be intimately linked to pain and the molecular basis of nociceptor signaling ^6^. TRPV1 activates a subset of slowly conducting thinly myelinated and unmyelinated somatosensory primary afferent neurons of the dorsal root ganglia (DRG) of the spinal cord. Our task here will be to briefly outline some of the major characteristics of TRPV1 activation established largely in DRG neurons and contrast these with recent developments in an entirely different set of TRPV1-expressing sensory afferents embedded deep within the body. In this article, I will focus on TRPV1 as a sensory transduction molecule and emphasize recent complementary work that suggests several new aspects of TRPV1 signaling in non-somatosensory primary afferents linked to innocuous neural functions.

## Key attributes of TRPV1

The first key intrinsic properties of TRPV1 as an ion channel to appreciate are inseparable: calcium and depolarization. The strong calcium preference means that TRPV1 essentially serves as a calcium channel. TRPV1 conducts nearly 10-fold more calcium ions than other cations, so that measures of intracellular calcium levels often substitute as a metric for TRPV1 activity ^1^. However, the inward current flow depolarizes and recruits voltage-activated channels exciting the neurons. A second important aspect of TRPV1 is gating, where three distinct stimuli are represented in separate sequences within the TRPV1 structure ^7, 8^. The three stimuli presumably produce conformation changes to the TRPV1 protein, resulting in gating the channel open, namely vanilloid binding to one site, protons binding to an additional acidic site, and raised temperature critically changing a third site ^9– 12^. Remarkably, these three distinct stimuli gate open TRPV1 channels singularly but can act cooperatively in combination to facilitate gating so that threshold temperatures are lower in the presence of vanilloid—a property of this single protein which makes it a multimodal integrator ^13, 14^. Singly, however, the minimum gating stimuli are notable for their extreme, supraphysiological intensities even at threshold: high temperature (>42 °C), low pH (~6), and vanilloid ligands such as capsaicin, a substance foreign to the mammalian body ^15, 16^. Physiologically, such stimulus levels are reached only under extraordinary circumstances most often associated with frank tissue damage. The high affinity of plant-derived substances for TRPV1 such as capsaicin and the picomolar affinity resiniferatoxin (RTX) raised the question of whether these compounds mimic endogenous vanilloid substances in vertebrate animals and humans ^17, 18^. Certainly, on the basis of comparative chemical similarities, vanilloid moieties have been suggested as endogenous agonists (endovanilloids) to TRPV1 ^19, 20^. The complex interrelationship of anandamide and arachidonate metabolites and their highly varied fates offers a wide range of agonistic possibilities as well as uncertainties in the natural context ^21– 23^. These properties of TRPV1 gating, ion selectivity, and modulation were remarkably conserved across a wide variety of experimental contexts, including heterologous expression systems such as transfected HEK293 cells and dispersed cultured DRG neurons to brain slices and *in vivo*. A third aspect of TRPV1 is the loss of responsiveness with continued activation. Prolonged or repeated exposure to high agonist concentrations substantially desensitizes TRPV1 function and depends on calcium entry and dephosphorylation ^24– 26^. In the extreme with neonatal animals, TRPV1 agonists selectively kill TRPV1-expressing sensory neurons likely due to calcium overload, whereas in adult animals, such treatments deplete neurotransmitter and damage primary sensory neurons reducing or eliminating function ^3, 27^. Intrathecal RTX produced analgesia as well as increases in blood pressure and heart rate ^28^. TRPV1 expression makes central axons vulnerable to damage by intrathecal RTX ^29^. Desensitization and competitive antagonism were exploited in attempts to design effective clinical analgesics ^30^. Whereas preclinical results were promising, the trial results were confounded by hyperthermia. Selective TRPV1 antagonists triggered a perplexing rise in body temperature largely through what appears to be block of a tonically activated peripheral TRPV1 site and this side effect curtailed clinical deployment as an analgesic ^31^. Note that these trials focused solely on addressing somatosensory nociceptors but simultaneous visceral afferent contributions to temperature regulation foiled these efforts. This remains a controversial topic and TRPV1 vagal afferents are implicated ^32^.

## TRPV1 in the broader context of primary afferents

The link between TRPV1 and nociception is clear and compelling. What often is overlooked is the presence of TRPV1 ^+^ non-somatosensory neurons. Extensive work links capsaicin with subpopulations of somatosensory afferents with slower-conducting axons (Aδ and C) beyond DRG neurons ^3, 6^. Peripheral TRPV1 and capsaicin actions extend to unmyelinated cranial viscerosensory neurons to activate neural pathways controlling innocuous but vital functions like gastrointestinal control ^33^, blood pressure ^34, 35^, and respiratory control ^36^. TRPV1 activation strongly excites cranial visceral afferents and activates pathways not involved in conscious perception but rather more in reflex alteration of autonomic functions, for example. These actions of capsaicin were described initially over 50 years ago ^3^ but depended on the activation of specific subsets of visceral or interoceptive sensory neurons that included strong cardiorespiratory ^37, 38^ and thermoregulatory ^39^ responses.

Immunostaining and blots revealed dense staining for TRPV1 in nodose ganglion but extended into the brainstem at the nucleus of the solitary tract (NTS) and implicated TRPV1 in vagal primary afferents and their central transmission in the TRPV1-expressing cohort of primary afferents ^40^. Interestingly, activation of these vagal central pathways inhibits nociceptive reflexes; that is, they are anti-nociceptive in consequence ^41^. The frequency and intensity response profiles for vagal nerve stimulation are consistent with C-fiber activation and inhibited dorsal horn nociceptive responses; thus, craniovisceral C-fiber activation inhibits the somatosensory nociceptive process in the spinal cord ^42^. Clearly, as both cranial and somatosensory afferents express TRPV1, this fact represents potential confounders in pain therapeutic strategies using vanilloid-based drugs. The relatively protected internal locations of visceral afferent TRPV1 limits heat and acid deviations to those compatible with life and thus are unlikely to reach canonical threshold levels in normal tissue. Such constraints raise interesting questions about the adequate stimulus for visceral and central TRPV1 endogenous activation.

## Localization of TRPV1 ^+^ neurons

Early tritiated RTX binding assays identified extensive central structures, some directly linked to primary sensory afferents but others not (olfactory nuclei, the cerebral cortex, dentate gyrus, thalamus, hypothalamus, periaqueductal grey, superior colliculus, locus coeruleus, and cerebellar cortex) ^35^. Recently, however, new investigations featuring highly sensitive genetic tracing approaches ^43– 46^ found a much more restricted distribution of TRPV1 markers limited chiefly to primary afferent neurons in the spinal cord DRG, the cranial nerve cervical ganglia, and the trigeminal ganglia. The highest density of TRPV1 expression included central primary afferent synaptic terminal fields. TRPV1 expression was limited to a few discrete brain regions, including near the caudal hypothalamus, but was absent in regions previously linked to TRPV1 pharmacologically such as the hippocampus ^46^. TRPV1 within primary afferents was limited largely to peptidergic, primary afferent neurons ^43^. Expression of TRPV1 is present throughout these neurons – peripheral sensory endings, axonally as well as the cell body and is not limited to specific locations within neurons such as proteins limited to synaptic regions or the spike initiation zone ^40^. However, we have only a limited understanding of the details of trafficking control. TRPV1 expression is increased during inflammation and the pro-inflammatory cytokine tumor necrosis factor-alpha promoted increased TRPV1 insertion into plasmalemma of cultured trigeminal primary afferent neurons in a process associated with co-expression of other synaptic proteins, including Munc18-1, syntaxin1, and SNAP-25 ^47^. It remains uncertain whether this scheme is generalizable and especially whether it differs between somatosensory and visceral primary afferents.

## TRPV1 in brainstem synaptic signaling

Craniovisceral primary afferent neurons (nodose, jugular, and petrosal) send axons centrally via the solitary tract (ST) to synapse predominantly within caudal portions of the NTS ^48, 49^. Most ST afferents have unmyelinated axons which overwhelmingly are TRPV1 ^+^, whereas the faster-conducting Aδ lack TRPV1 ^50^. Despite such expression, endogenous TRPV1 activity is difficult to discern in a largely homeostatically controlled internal milieu. *In vivo* evidence of endogenous TRPV1 activation in cranial afferents is indirect and controversial ^30, 32, 51, 52^ and suggests that tonic TRPV1 activation of vagal afferents contributes to thermoregulation. Similarly, the activation of TRPV1 on central sensory terminals seems unlikely given the high canonical threshold requirements ^2^. ST afferent transmission to second-order NTS neurons is relatively conventional with significant deviations from central glutamate transmission expectations. ST axons release glutamate to generate large excitatory postsynaptic currents (EPSCs) primarily through non-NMDA receptors as well as modulation via metabotropic glutamate receptors (mGluRs) ^53– 55^. The base, uniformly high probability of glutamate release ^56– 58^ means that frequency-dependent depression dominates afferent transmission. Stimuli delivered to the visible ST activate tract axons generating all-or-nothing EPSCs consistent with unitary responses with high likelihood of triggering postsynaptic action potentials. Such ST-evoked transmission relies on the synchronous release of glutamate vesicles. ST input is most often limited to a single afferent, suggesting that convergence is quite limited ^58^ as observed *in vivo* ^59^. Remarkably, even when multiple ST inputs do converge on a single neuron, ST inputs either are TRPV1 ^+^ or lack TRPV1—a segregation of afferents by TRPV1 expression regardless of sensory modality ^60– 62^.

Synaptic responses suggest that presynaptic glutamate release mechanisms within TRPV1 ^+^ ST terminals differ from TRPV1 ^−^ terminals not by synchronous release ^58^ but by two additional modes of glutamate release. Rates of “spontaneous” EPSCs (that is, action potential–independent release) are 10-fold higher than TRPV1 ^−^ afferents even in tetrodotoxin (TTX) ^63, 64^. The second added mode of release is the appearance of “asynchronous” spontaneous EPSCs (sEPSCs) following bursts of ST stimuli. Asynchronous release was elevated for seconds following the cessation of ST-evoked release. With sustained high frequencies of ST activation, synchronous EPSC amplitudes declined to less than 15% of control, but paradoxically the asynchronous rate simultaneously rose nearly fourfold ^64^. Thus, the probability of evoked release declined while asynchronous release rose, indicating that separate mechanisms controlled distinct pools of glutamate vesicles ^64^. Synchronous and asynchronous release required calcium entry through voltage-activated calcium channels (VACCs), but sEPSCs were unaffected by block of N-type VACCs ^65^. Thus, two modes of glutamate release depended on VACC calcium entry but spontaneous release did not. ST transmission depended on multiple, non-overlapping pools of vesicles. But how was calcium being separated within these terminals and where was calcium for sEPSCs coming from?

TRPV1 expression was clearly limited to ST central terminals within the NTS and predicted elevated sEPSC rates ^46, 66, 67^. Capsaicin (50 to 100 nM) had two effects on ST transmission: it robustly increased “spontaneous” glutamate vesicle release (sEPSCs), but within 1 to 5 minutes of exposure, ST shocks fail to activate the synchronized vesicle release for evoked EPSCs despite the continued high rate of sEPSCs. This puzzling pairing of facilitating one release while inhibiting the other reflects the dual nature of TRPV1 activation: depolarization and calcium entry, respectively. ST-evoked transmission required intact excitability to convey the ST shocks and excitation from axon site to terminal. We attribute the blockade of ST-evoked release during TRPV1 to sustained depolarizing inward currents remote from the terminals which inactivate voltage-dependent excitability as reflected in gradual increases in conduction time (ST-EPSC latency) preceding synaptic failure ^68– 70^. Calcium entry via TRPV1 selectively increases sEPSC rate.

Neurotransmission depends most directly on calcium ^71^. We anticipated that, since TRPV1 is highly calcium-selective, TRPV1 activation would augment glutamate release. Certainly, reducing external calcium concentration decreased the sEPSC rate ^70^, so calcium entry is required. In TTX, cadmium, a broad-spectrum VACC blocker, blocked evoked release without altering sEPSCs in TRPV1 ^+^ neurons—findings that collectively indicate that VACCs do not contribute to spontaneous release of glutamate. However, for NTS neurons, lowering the bath temperature to room temperature reduced sEPSCs and raising the temperature elevated sEPSC rate only in TRPV1 ^+^ neurons but neurons with ST inputs lacking TRPV1 were unaffected by such temperatures ^63– 65^. Thus, physiological temperatures were gating presynaptically expressed TRPV1 to allow calcium entry and produced a basal, stochastic vesicle release onto NTS second-order neurons. Vanilloid and temperature act cooperatively at ST TRPV1 to sensitize basal glutamate release ^69^. Temperature affected ST conduction times irrespective of TRPV1 expression but did not alter ST-EPSC amplitudes (that is, release) ^69^. Interestingly, this also indicated that TRPV1-related calcium influx into the synaptic terminals did not contribute to synchronous release. Together, the evidence suggested that TRPV1 controlled a distinct pool of glutamate vesicles that were not affected by action potentials. The independence of ST-EPSCs from TRPV1 activity may mean that the vesicles released by each stimulus are somehow effectively isolated from each other.

Multiple potential sources of calcium entry exist in ST terminals and include a diverse VACC family ^72^ plus TRPV1 and serotonin 3 receptors (5-HT _3_Rs) ^71^. Logically, calcium entering ST terminals might mix to augment multiple forms of glutamate release. Already, augmented TRPV1 activity did not boost ST-evoked EPSCs as would be expected. We decided to introduce calcium buffers to intercept calcium entering via different calcium entry sources simultaneously ^73, 74^. Our advantage with ST transmission is that we can assay multiple modes of synaptic release (evoked, spontaneous, and asynchronous) simultaneously within individual neurons. Depending on affinity and concentration, calcium buffers will intercept calcium diffusing from the entry pore to the vesicle so that differences in buffering magnitude and timing will reflect positioning of calcium source relative to released vesicles. The introduction of calcium buffer first reduced asynchronous EPSC rates, suggesting that asynchronous vesicle release required the greatest diffusion distances compared with synchronously released vesicles making up the evoked ST-EPSCs amplitudes ^65^. Interestingly, sEPSCs or thermal responses were unaltered by buffering, suggesting a close association of TRPV1 with its vesicles within a nanodomain. Buffering therefore distinguishes asynchronous vesicles as possessing a highly sensitive calcium sensor located perhaps more distant from VACCs than synchronous vesicles. Taken as a whole, our findings suggest distinct mechanisms of release for synchronous, asynchronous, and spontaneous vesicles that are likely to reside in unique, spatially separated vesicle domains. Collectively, these studies support a critical topology within the terminal that isolates a TRPV1 mechanism of release separate from a VACC release mechanism ^71^. Such a nanostructuring of the synaptic terminals offers a scaffold for differential release, modulation, and integration.

## Modulation of release modules

G protein–coupled receptors (GPCRs) encompass a wide swath of signaling molecules and a substantial genomic proportion ^75, 76^. NTS and specifically the presynaptic afferent terminals are richly endowed with more than a score of different GPCRs often devoted to peptides ^48, 49^. Given the distinct release patterns centered on calcium entry sources and release modules, ST-NTS transmission offers diverse examples of both highly focused and broad-based GPCR targeting. Perhaps the most discrete example to date is CB1. The majority of ST inputs expressed CB1 regardless of TRPV1 responsiveness. Activation of CB1 selectively inhibits evoked synchronous glutamate release without altering basal or thermally activated TRPV1-mediated release of glutamate ^77^. The bifunctional, endogenous arachidonate metabolite N-arachidonyldopamine (NADA) activated both CB1 and TRPV1 and appropriately inhibited evoked ST-EPSCs while augmenting sEPSCs with each effect blocked by highly selective, competitive antagonists targeted to either CB1 or TRPV1 ^77^. Activation of vasopressin 1a receptors (V1aR), depressed both spontaneous and ST-evoked synchronous release of glutamate and independently blocked conduction into ST terminals ^56^. Activation of oxytocin receptors (OTRs) increased both spontaneous and ST-evoked synchronous release of glutamate ^57^. Thus, V1aR and OTR are expressed in small subsets of ST afferent terminals and likely represent highly discrete modulation of specific afferent pathways through NTS and beyond. Given the block of both spontaneous and evoked release, these GPCRs must be expressed at both release nanodomains controlling those subsets of vesicles. One of the most ubiquitous GPCRs in NTS is ST presynaptic gamma amino butyric acid type B receptor (GABA _B_R). GABA _B_Rs depressed all forms of glutamate release in NTS: synchronous, asynchronous, and spontaneous, including thermal gating of TRPV1 ^78^. We speculate that the patterns suggest that GPCRs will be localized within specific release module nanodomains within ST terminals. The implication is that highly discrete modulation relies on physical distribution whereas broad GPCR modulation reflects multiple placements of GPCRs within or across signaling domains. Surprising genetic-proteomic evidence suggests that, in some peripheral somatic sensory neurons, GABA _B_R 1 subunits are positioned with TRPV1 and can switch the sensitized state of TRPV1 and this non-canonical, direct coupled mechanism requires a critical proximity ^79^. Suffice it to say, the nanoscale landscape regarding TRPV1 will require improved tools and more refined spatially discrete approaches to verify the key aspects of nanoscale signaling and appropriate trafficking to support such mechanisms.

## Out-of-canon TRPV1

The experimental results in NTS and brainstem to date are importantly discrepant with multiple canonical assumptions. First and foremost, the basal thermal threshold for TRPV1 lies in the mid-range of physiological temperatures existing at the expression site. *In vivo*, introduction of capsazepine alone systemically or into NTS had no effect on respiration, blood pressure, or heart rate ^80, 81^. Although temperature is set at artificially low temperatures for slice recordings, the sEPSC rates average over 20 Hz at normal brain temperatures and generate substantial autonomous action potential firing without ST stimulation ^63, 64^. Clearly, such autonomous firing and the signals arriving at distant projection sites could be modulated by GPCRs without the necessity for peripheral afferent activity. The sustained activation of ST TRPV1 by vanilloids or temperature suggests that vesicle depletion or desensitization is moderate to none over extended time frames ^64, 69^. Vanilloid competitive antagonists are effective in blocking agonist (capsaicin or RTX) responses at ST TRPV1 but not other modes of TRPV1 activation, including heat in NTS ^69^, in contrast to capsazepine block of heat responses in transfected HEK293 cells ^40^. Externally applied QX-314 has attracted considerable attention in blocking nociception when coupled to TRPV1 activation in somatosensory neurons ^82, 83^, but QX-314 non-selectively blocks ST-NTS transmission regardless of TRPV1 expression ^68^. Collectively, such contrasting results raise the question of whether somatosensory TRPV1 is fundamentally different from craniovisceral TRPV1. Developmentally, DRG and nodose neurons derive from neural crest and placode cells, respectively, and these embryological origins are associated with a number of distinctions regarding ion channel, GPCR, and neurotransmitter expression ^84– 86^. Although the origins of these functional differences remain unclear, the evidence of differences is substantial.

## Endogenous activation

The origin of the sensitized or physiological range of thermal sensitivity of cranial visceral TRPV1 afferents is unclear. In ST afferents, sEPSC release is insensitive to competitive vanilloid antagonists, suggesting that, *in vitro*, an endogenous agonist (for example, inflammatory mediator) is not responsible ^69^. Suggested endogenous ligands for TRPV1 include anandamide and other bioactive lipids like lysophosphatidic acid ^87^, but functional cases are limited ^88^. Oleoylethanolamide (OEA) is an endogenous fatty acid ethanolamine that is produced in the intestinal mucosa in the fed state. High concentrations of OEA activate vagal afferents ^89^, but it is unclear whether endogenous levels are sufficient. Oleic acid inhibits DRG TRPV1 ^90^, as does anandamide ^91^, but neither is effective in ST TRPV1 ^92^ (unpublished results). Thus, the role of endogenous lipid metabolites in TRPV1 activation or sensitization remains largely unresolved.

## Future directions

TRPV1 is long recognized as an impactful physiological and pathophysiological molecule. The bluntness and imprecision of many of the available tools have been remarkably frustrating. Drug development has been stymied by targeted purpose programs that were stopped by unintended TRPV1 actions off target. Clearly, newer and perhaps more discrete approaches and tools might be helpful in all respects. The complicated fate of membrane lipid metabolites and their spatial control make the pursuit of endogenous TRPV1 actors daunting. Biology-informed synthetic chemistry may hold promise ^22^. For example, synthesis of appropriately spatially constrained, photoactivated tools might offer better insights into discrete localization of TRPV1 in appropriate biological models ^93^.

## Abbreviations 

DRG, dorsal root ganglia; EPSC, excitatory postsynaptic current; GABA _B_R, gamma amino butyric acid type B receptor; GPCR, G protein–coupled receptor; NTS, nucleus of the solitary tract; OEA, oleoylethanolamide; OTR, oxytocin receptor; RTX, resiniferatoxin; sEPSC, spontaneous excitatory postsynaptic current; ST, solitary tract; TRPV1, transient receptor potential vanilloid 1; TTX, tetrodotoxin; V1aR, vasopressin 1a receptor; VACC, voltage-activated calcium channel
